# Multi-dimensional optimization of In_0.53_Ga_0.47_As thermophotovoltaic cell using real coded genetic algorithm

**DOI:** 10.1038/s41598-021-86175-5

**Published:** 2021-04-08

**Authors:** Mansur Mohammed Ali Gamel, Pin Jern Ker, Hui Jing Lee, Wan Emilin Suliza Wan Abdul Rashid, M. A. Hannan, J. P. R. David, M. Z. Jamaludin

**Affiliations:** 1 Institute of Sustainable Energy, Universiti Tenaga Nasional, 43000 Kajang, Selangor Malaysia; 2 Institute of Power Engineering, Universiti Tenaga Nasional, 43000 Kajang, Selangor Malaysia; 3 Department of Electronic and Electrical Engineering, The University of Sheffield, Firth Court, Western Bank, Sheffield, S10 2TN UK

**Keywords:** Solar energy and photovoltaic technology, Thermophotovoltaics

## Abstract

The optimization of thermophotovoltaic (TPV) cell efficiency is essential since it leads to a significant increase in the output power. Typically, the optimization of In_0.53_Ga_0.47_As TPV cell has been limited to single variable such as the emitter thickness, while the effects of the variation in other design variables are assumed to be negligible. The reported efficiencies of In_0.53_Ga_0.47_As TPV cell mostly remain < 15%. Therefore, this work develops a multi-variable or multi-dimensional optimization of In_0.53_Ga_0.47_As TPV cell using the real coded genetic algorithm (RCGA) at various radiation temperatures. RCGA was developed using Visual Basic and it was hybridized with Silvaco TCAD for the electrical characteristics simulation. Under radiation temperatures from 800 to 2000 K, the optimized In_0.53_Ga_0.47_As TPV cell efficiency increases by an average percentage of 11.86% (from 8.5 to 20.35%) as compared to the non-optimized structure. It was found that the incorporation of a thicker base layer with the back-barrier layers enhances the separation of charge carriers and increases the collection of photo-generated carriers near the band-edge, producing an optimum output power of 0.55 W/cm^2^ (cell efficiency of 22.06%, without antireflection coating) at 1400 K radiation spectrum. The results of this work demonstrate the great potential to generate electricity sustainably from industrial waste heat and the multi-dimensional optimization methodology can be adopted to optimize semiconductor devices, such as solar cell, TPV cell and photodetectors.

## Introduction

In recent years, thermophotovoltaic (TPV) has been escalating as a promising technology for high power density generation. A TPV system converts thermal radiations from combustion of fuels, industrial waste heat, or nuclear energy into electricity. The advantages of noiseless, high reliability, mechanically stable without moving parts, and large power density, make TPV suitable for a vast range of real-world applications such as electrical generator^1–3^, aerospace applications^1,4^, submarine^5^, vehicle^6^, solar thermophotovoltaic (STPV) cell^7–9^, energy storage^10,11^, waste heat recovery system in metal-alloy industries^2,12,13^, power plant^14,15^ and fuel cell^16^. The TPV converters mainly utilize narrow bandgap (NB) semiconductor materials which allow them to harvest the maximum amount of infrared radiations (IRs). The advancement of nanotechnology and material science since 1990s have boosted the development of various NB TPV cells, such as germanium (Ge)^17^, indium arsenide (InAs)^18^, gallium antimonide (GaSb)^19^, indium gallium arsenide (InGaAs)^20^, indium gallium antimonide (InGaSb)^21^, indium gallium arsenide antimonide (InGaAsSb)^22^ and indium arsenide antimonide phosphate (InAsSbP)^23^. In the last 3 decades of research in TPV, most researchers focus on the utilization of GaSb cell due to its narrow bandgap of 0.72 eV. US company JX Crystals Inc has developed a high-performance GaSb TPV cell which is commercially available and is widely used in various TPV systems^24^. The GaSb TPV cell was reported to have an efficiency of 29% under radiation temperature of 1548 K^13^. On the other hand, InGaAs, which has similar bandgap energy and potential in achieving high TPV performance, was relatively more common for applications in telecommunication and sensing. In_1-x_Ga_x_As is a ternary semiconductor with bandgap energy (*E*_*g*_) that can be engineered from 1.42 to 0.36 eV by varying the *x* composition of Ga atom, which corresponds to cutoff wavelengths *(λ*_*c*_) from 0.87 to 3.34 µm^25^. At *x* = 0.47, In_0.53_Ga_0.47_As semiconductor material can be grown lattice-matched on an indium phosphide (InP) substrate, corresponds to *E*_*g*_ and *λ*_*c*_ of 0.74 eV and 1.68 µm, respectively. Moreover, In_0.53_Ga_0.47_As is a promising TPV cell due to its high crystal quality and the cost-effectiveness of InP substrate, making it suitable for large scale production as compared to other TPV materials.

It is worth mentioning that the existing epitaxy growth technology of metal–organic vapor-phase epitaxy (MOVPE) has the ability to produce In_0.53_Ga_0.47_As/InP heterojunction with high crystal quality and low defect density^26,27^. The main structure of In_0.53_Ga_0.47_As configuration includes the emitter, base, front surface field (FSF), back surface field (BSF), cap and buffer layers. In previous literature, the base thickness was reported between 1 and 5 µm, and emitter thickness was between 0.05 and 0.44 µm^27–29^. Several structures reported the use of highly doped In_0.53_Ga_0.47_As cap layer ~ 1 × 10^19^ cm^−3^ and highly doped InP BSF/buffer layer ≥ 1 × 10^18^ cm^−3^. Table 1 reviews the design structure and output performances of In_0.53_Ga_0.47_As photovoltaic (PV) cell under different testing conditions. Typical In_0.53_Ga_0.47_As cells have open-circuit voltage (*V*_*oc*_), short circuit current density (*J*_*sc*_), fill factor (*FF*) and efficiency (*η*) ranging between 0.26 and 0.45 V, 18.8 and 64.5 mA/cm^2^, 59 and 74.2%, and 4.2 and 14.37%, respectively under air-mass 0 (AM0) and air-mass 1.5 (AM1.5) illuminations^26,28,30,31^. In 2019, Omair et al.^32^ reported a TPV cell efficiency of 29.1% under 1480 K radiation temperature, by recycling sub bandgap photons to the radiator. It is possible to achieve > 50% cell efficiency by improving the series resistance, material quality and reflectivity using chamber with high mirror reflectivity. Recently, Fan et al.^33^ presented an air-bridge structure that enables photons recycling, increasing the reflection of sub-bandgap photons to 99% and enhancing the efficiency up to 30%. As summarized in Table 1, the TPV testing conditions resulted in higher output performance as compared to solar spectrums. Moreover, commercially available TPV cell has the advantage of producing high output power density, ~ 26 times higher than the output power of solar PV cell^34,35^. All the reported work in the optimization of In_0.53_Ga_0.47_As cell are based on the alteration of single design variable^36,37^. Recently, a multi-variable optimization of solar cell was used to optimize the physical properties of electron transport materials, hole transport materials, and metal contact and layers thickness. It was accomplished using the MATLAB optimization toolboxes incorporated with one-dimensional (1D) Solar Cell Capacitance Simulator (SCAPS) for device simulation^40^. However, the study did not take into account the impact of doping concentration on the cell performance.

**Table 1:** Structure design and the performance of reported heterojunction In_0.53_Ga_0.47_As cell.

Str	Cap thickness (doping)	FSF thickness (doping)	Emitter thickness (doping)	Base thickness (doping)	BSF thickness (doping)	Buffer thickness (doping)	*V*_*oc*_ (V)	*J*_*sc*_ (mA/cm^2^)	*FF* (%)	*η* (%)	Condition
p-n^33^	n/a	0.02 (1 × 10^18^)	none	1 (1 × 10^17^)	0.0 (1 × 10^18^)	n/a	0.425	336.76	74.47	31.3	1473 K (photon recycling)
n-p^26^	0.3 (n/a*)	0.05 (n/a)	0.3 (n/a)	3 (n/a)	0.25 (n/a)	none*	0.35	57.7	71.2	14.37	AM1.5
n-p^32^	0.2 (1 × 10^18^)	0.02 (1 × 10^18^)	none	2.5 (1 × 10^17^)	0.1 (1 × 10^18^)	0.2 (1 × 10^18^)	0. 529	918	73	29.1	1480 K (Photon recycling)
(n/a)^38^	n/a	n/a	n/a	n/a	n/a	n/a	~ 0.225	0.5	58	16.4	1323 K
n-p^39^	0.025 (1 × 10^19^)	0.1 (2 × 10^18^)	0.1 (5 × 10^17^)	2.5 (2 × 10^17^)	0.3 (1 × 10^18^)	with BSF	0.405	288	65	12.4	0.62 W/cm^2^ Tungsten– halogen lamp (3250 K)
p-n^39^	0.025 (1 × 10^19^)	0.1 (7 × 10^18^)	2 (1 × 10^17^)	0.3 (5 × 10^17^)	0.3 (1.5 × 10^18^)	with BSF	0.419	284	62	12.1	
n-p^30^	n/a	n/a	0.3 (n/a)	3 (n/a)	n/a	1 (1 × 10^19^)	0.3–0.31	21.5– 24.9	66–70	12.9–13.6	AM1.5
n-p^36^	n/a	n/a	0.1 (3 × 10^17^)	2 (8 × 10^16^)	n/a	n/a	0.38 and 0.44	5 × 10^1^ and 6 × 10^2^	n/a	15 and 18	4000 K (0.1 and 1 W/cm^2^)
n-p^40^	none	400 (n/a)	n/a (1 × 10^19^)	2–4 ((1–5) × 10^17^)	none	n/a	~ 0.45–0.48	~ 4 × 10^3^	~ 69–72.5	~ 13.7–15	1800 K
p-n^41^	0.1 (1 × 10^19^)	0.07 (1 × 10^18^)	0.25 (1 × 10^19^)	1 (undoped)	0.5 (1 × 10^18^)	n/a	0.341	43.1	68	10.11	AM1.5
n-p^28^	n/a	0.05 (1 × 10^18^)	0.4 (1 × 10^18^)	3 (4 × 10^17^)	0.4 (2 × 10^18^)	none	0.4	45.1	66.9	12.1	AM1.5
n-p^42^	0.3 (1 × 10^19^)	0.05 (1 × 10^18^)	0.4 (1 × 10^18^)	3 ((1–4) × 10^17^)	0.1 (1 × 10^18^)	none	0.39	42.8	71	11.8	AM1.5
p-n^43^	0.1 (1 × 10^19^)	0.1 (2 × 10^18^)	0.3 (1 × 10^19^)	2 (5 × 10^17^)	0.1 (1 × 10^18^)	1 (2 × 10^19^)	0.399	56.4	71.5	11.7	AM0
n-p^44^	0.3 (1 × 10^19^)	0.05 (1 × 10^19^)	0.1 (1 × 10^19^)	4 (1 × 10^17^)	2 (1 × 10^19^)	none	0.303	9.4	n/a	~ 1.36	1273 K

n/a means no data available and none* represents the unused layer.

The optimization of TPV cell structure is critical in getting the highest achievable *η*. A slight increment in *η* will significantly increase the output power and total energy. The simplest method to optimize a structure is the single-layer/variable optimization, which optimizes only a single parameter at a time while other parameters are kept constant. Several attempts were made to optimize single variable, especially on the emitter and base layers of In_0.53_Ga_0.47_As cell^36,37^. However, device performance depends collectively on all the design variables^45,46^, and a more heuristic optimization that considers the effect of all important variables for the In_0.53_Ga_0.47_As TPV cell is necessary to achieve the optimum cell efficiency. Therefore, this study investigates the effect of each variable through single variable optimization and performs multi-dimensional (simultaneous multi-variables) optimization using real coded genetic algorithm (RCGA) to obtain the optimum configuration of In_0.53_Ga_0.47_As TPV cell.

## Methods

### In_0.53_Ga_0.47_As cell modelling and validation

**Figure 1:**
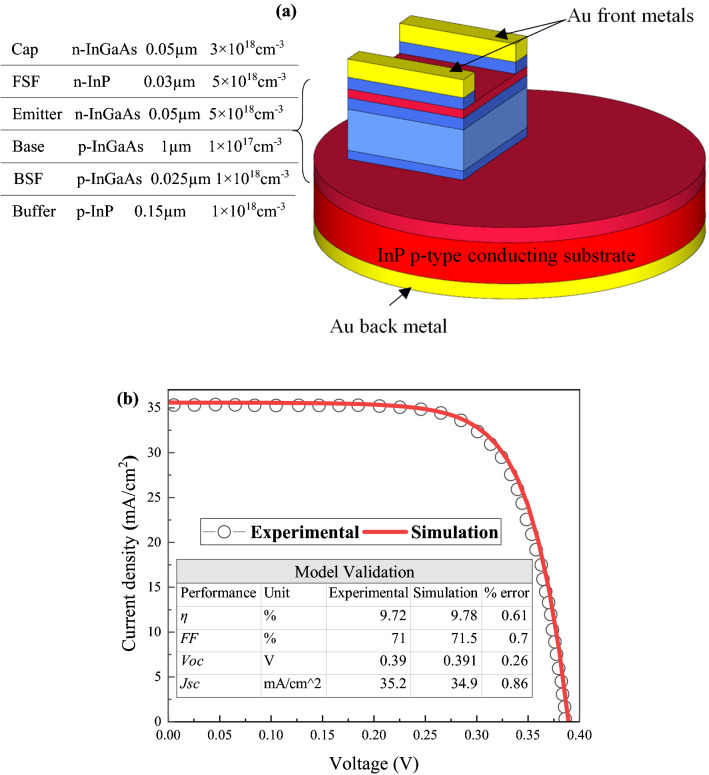
Simulation to reported experimental of In_0.53_Ga_0.47_As (**a**) Baseline n-p structure^27^; the drawing was created using SketchUp^58^ (**b**) JV curves and performance parameters.

The In_0.53_Ga_0.47_As cell was modeled using the computational numerical modeling TCAD Silvaco ATLAS software package. A 2-dimensional (2D) In_0.53_Ga_0.47_As model was constructed using DevEdit tool, while the computations on the electrical characteristics were mainly performed with ATLAS. The input–output transformation method is used to validate the In_0.53_Ga_0.47_As simulation model with similar experimental work reported by Sodabanlu et al*.*^27^. The input parameters are structure design and testing conditions. The structure design includes front and back gold contacts, thickness and doping concentration of the emitter, base, BSF, FSF, buffer and cap layers, as shown in Fig. 1a. No anti-reflective coating (ARC) was considered in the structure. The validation testing is performed under a standardized solar spectrum AM1.5 at room temperature (300 K). The output performance parameters that are of primary interest include JV-curve, *J*_*sc*_, *V*_*oc*_, *FF* and *η*. The simulation took into consideration the Auger, radiative and Shockley–Read–Hall (SRH) recombination as well as the carrier’s lifetime and mobility concentration-dependent models. At a doping concentration of 1 × 10^17^ cm^−3^, lifetime of 16 ns for electrons and 40 ns for holes were used based on the model. While the In_0.53_Ga_0.47_As model and testing conditions remain constant over the validation process, the materials parameters of the In_0.53_Ga_0.47_As and InP were varied within the reported range in previous literature. Table 2 summarizes the electrical properties of In_1-x_Ga_x_As as a function of *x* composition and concentration-dependent models.

**Table 2:** Material parameters of In_1-x_Ga_x_As and In_0.53_Ga_0.47_As at 300 K.

Material properties at 300 K	(*x*) is the composition and (*N*) is the doping level	Range	Numerical values determined in this work
Bandgap^26,47,48^ *E*_*g*_ (eV)	$E_{g} (x) = 0.436x^{2} + 0.629x + 0.39$	0.734–0.77	0.75
Affinity^26,47,48^ *E*_*ea*_ (eV)	$E_{ea} (x) = 4.9 - 0.83x$	4.47–4.5	4.47
Permittivity^26,47^ *ε*	$\varepsilon (x) = 0.67x^{2} - 2.87x + 15.1({\text{static}})$ $\varepsilon (x) = 12.3 - 1.4x({\text{high frequency}})$	11.6–13.899	13.8
Electron density of states^26,47^ *N*_*c*_ (cm^−3^)	$$N_{c} (x) = 2.289 \times 10^{17} x^{2} + 1.541 \times 10^{17} x + 8.7 \times 10^{16}$$$$N_{c} (x) = 2(\frac{{2\pi m_{e} \times kT}}{{h^{2} }})^{3/2}$$	2.1 × 10^17^	2.1 × 10^17^
Hole density of states^26,47^ *N*_*v*_ (cm^−3^)	$$N_{v} (x) = 1.124 \times 10^{17} x^{2} + 2.288 \times 10^{18} x + 6.6 \times 10^{18}$$$$N_{v} (x) = 2(\frac{{2\pi m_{h} \times kT}}{{h^{2} }})^{3/2}$$ $${\text{I}} = {\text{I}}_{0} 10^{{ - \varepsilon {\text{cl}}}}$$	7.7 × 10^18^	7.7 × 10^18^
Electron mobility^26,49–52^ $$\mu$$_e_ (cm^2^ V^−1^ s^−1^)	$$\mu_{e} (x) = (40 - 80.7x + 49.2x^{2} ) \times 1000$$ $$\mu_{h} \cong 300/400$$$$\mu_{e,h} (N) = \mu_{e,h(\min )} + \frac{{\mu_{e,h(\max )} - \mu_{e,h(\min )} }}{{1 + (N_{D,A} /N_{ref(e,h)} )^{Z} }}$$	3372–14,000	$$\mu$$_max_ = 11,599 $$\mu$$_min_ = 3372 *N*_*ref*_ = 2.1 × 10^17^ cm^−3^ *z* = 0.76
Hole mobility^26,49–52^ $\mu$_h_ (cm^2^ V^−1^ s^−1^)		10–331	$\mu$_max_ = 331 $\mu$_min_ = 75 *N*_*ref*_ = 7.7 × 10^18^ cm^-3^ *z* = 1.37
Electron lifetime^26,49,50,53–57^ $$\tau_{e}$$. (s)	$$\tau_{e,h} (N) = (2.11 \times 10^{4} + 1.443 \times 10^{ - 10} N + 8.1 \times 10^{ - 29} N^{2} )^{ - 1}$$$$\tau_{e,h} (N) = \frac{{\tau_{0(e,h)} }}{{1 + (N_{D,A} /N_{ref(e,h)} )^{\gamma } }}$$ $$R_{SRH} = \frac{{pn - n_{i}^{2} }}{{\tau_{n} \left( {p - n_{i} } \right) + \tau_{p} \left( {n - n_{i} } \right)}}$$	0.05 × 10^–9^–55 × 10^–6^	$$\tau$$_o_ = 16 × 10^–9^ $$\gamma$$ = 0.73 *N*_*D*_ = 1 × 10^17^ cm^−3^
Hole lifetime^26,49,50,53–57^ $\tau_{h}$ (s)		0.1 × 10^–9^–90 × 10^–6^	$\tau$_o_ = 40 × 10^–9^ $\gamma$ =1.2 *N*_*A*_ = 1 × 10^17^ cm^−3^
Auger recombination ^26,48,49,53,55^ *R*_*Aug*_	$R_{Aug} = (C_{n} + C_{p} p)(np - n_{i}^{2} )$	2 × 10^–28^–8 × 10^–29^ 3.2 × 10^–28^–7 × 10^–29^	*C*_*n*_ = *C*_*p*_ = 8.1 × 10^–29^ cm^−6^ s^−1^
Radiative recombination ^26,48,49,55^ *R*_*Rad*_	$R_{Rad} = \frac{C}{4}(np - n_{i}^{2} )$	0.96 × 10^–10^–9.6 × 10^–11^	*C* = 0.96 × 10^–10^ cm^3^/s

^*^z represents mobility fitting parameter, ^*^$\gamma$ represents lifetime fitting parameter, ^*^R_*SRH*_ is SRH recombination, ^*^C_*rad*_ is electron and hole radiative coefficient, ^*^C_*n,p*_ are electron and hole radiative coefficient, ^*^C is auger coefficient, ^*^n is hole densities, ^*^p represents hole densities, *N*_*D,A*_ doner and accepter doping concentration and ^*^n_*i*_ represents intrinsic region.

Using the identified material parameters in Table 2, the current density–voltage (JV) characteristics of the In_0.53_Ga_0.47_As cell are obtained. The generated JV characteristics of the simulation model can be seen in Fig. 1b. It can be observed that a close agreement was obtained between the performance parameters of the simulation model and the reported experimental data. A percentage error of less than 1% was achieved for each parameter. For example, a percentage error of 0.61% between the experimental and simulation results was calculated for *η*. In addition, it was reported by other literature that the performance parameters of *η*, *FF*, *V*_*oc*_ and *J*_*sc*_, under similar testing condition (AM1.5), were in the range of 9.3–12.9%, 68–71%, 0.31–0.39 V and 21.5–42.8 mA/cm^2^, respectively^30,41,42^. The simulation results for the validation work achieved in this study are within the reported range; hence, validate the In_0.53_Ga_0.47_As cell model in this work.

### Single layer/variable optimization

A single variable optimization gives an indication of the significance of a variable to the TPV cell performance, reveals the trend of performance variation for each variable and more importantly it identifies the range of the design parameters accurately, and that provides the RCGA with a much faster convergence speed as well as a higher solution accuracy. Based on the reported values for the thickness and doping concentration of each layer in Table 1, their maximum and minimum values were first estimated. However, since the majority of the In_0.53_Ga_0.47_As structures are used for PV application, several simulations were conducted to modify the upper and lower boundary conditions to suit TPV testing conditions. Table 3 shows the range of the design parameters for the In_0.53_Ga_0.47_As TPV cell. Individually, each variable was manipulated while the rest of the design parameters in Fig. 1a remained constant. In the BSF analysis, base thickness was fixed at 10 µm to reduce the absorption in BSF, thereby allowing the investigation of the layer functionality as field generator. The optimization process was conducted under radiation temperatures from 800 to 2000 K at 50% beam illumination intensity. The 50% beam intensity was preferred as selective radiator usually emitted ~ 50% less power than an ideal blackbody^59^. In addition, a low pass optical filter at 2 µm was employed in the simulation following the reported TPV system^60–62^. Fourspring et al.^63^ illustrated the use of the edge short-pass filter and emphasized that the optical interference material has high spectral efficiency which reduces the amount of energy that reaches the TPV cell for wavelengths higher than 2 µm. The cell temperature was maintained at 300 K, assuming that it is controlled using an effective thermal management system^64^. The consideration of heat transfer between the TPV cell and the environment can be a more crucial subject of investigation for TPV devices with nano-gap between the device and the heat emitter^65^.

**Table 3:** The range of thicknesses and doping concentrations for the single variable optimization In_0.53_Ga_0.47_As structure.

In_0.53_Ga_0.47_As cell	Thickness range (µm)	Doping concentration range (cm^−3^)
Cap layer	0.01–0.3	1 × 10^16^–5 × 10^20^
FSF layer	0.01–0.3	1 × 10^16^–5 × 10^20^
Emitter layer	0.02–0.3	1 × 10^16^–1 × 10^19^
Base layer	1–30	5 × 10^16^–1 × 10^19^
BSF layer	0.02–1	1 × 10^16^–5 × 10^19^
Buffer layer	0.02–3	1 × 10^16^–1 × 10^20^

### Multi-dimensional optimization using real coded genetic algorithm method

**Figure 2:**
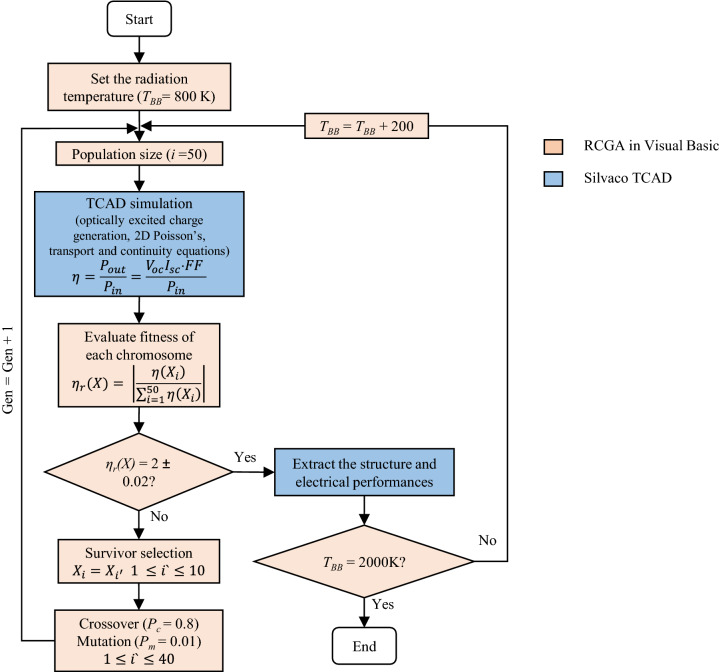
The flowchart that illustrates the hybridization of Silvaco TCAD with Real coded genetic algorithm.

The multi-dimensional optimization performs complete iterations for all possible combination of different variables to obtain the optimum values for all variables that achieve the highest efficiency. A flow chart of the multi-variable optimization of In_0.53_Ga_0.47_As structure under different radiation temperatures is presented in Fig. 2. The optimization process consists of both the device simulation module and the numerical optimization. Firstly, the device simulation module, where ATLAS was used to simulate the 2D In_0.53_Ga_0.47_As model. An initial population size of 50 input vector *X* was implemented in the final optimization version. It was demonstrated that a higher population size improves the accuracy and reduces the number of generation (Gen) required to allow the iterations to converge to the optimum In_0.53_Ga_0.47_As configuration. *X* is given in Eq. (1) as the six-layer/twelve design variables to be optimized.

1 $$X = X_{i} = [x_{i1} ,x_{i2} , \ldots ,x_{in} ] = [x_{i1} ,x_{i2} , \ldots ,x_{i12} ]$$

where *i* = 1–50 is the initial population size, *n* = 1–12 is the design variables based on some lower and upper physical constraints ${x}_{L}\le {x}_{in}\le {x}_{U}$ in Table 4. DBinternal tool is used to interfaced 50 sets of *X* to Deckbuild ATLAS, then solves the optically excited charge generation, 2D Poisson’s Equation, transport Equation, and continuity Equations and calculates the efficiency (*η*(*X*)) of the cell based on the inputs. Moreover, the numerical optimization of RCGA method was performed. Real coded is a direct representation of the variables, where no coding and encoding is required^66^. The objective function is to maximize the *η* of the In_0.53_Ga_0.47_As TPV cell, as presented in Eq. (2).

2 $$\eta_{\max } = \mathop {\max }\limits_{{x_{L} \le x_{in} \le x_{U} }} \eta (X)$$

where *η* is^20,67^:

3 $$\eta = \frac{{P_{out} }}{{Q_{emit} - Q_{filter} }}$$

**Table 4:** The variables upper and lower boundary conditions for the multi-dimensional optimization In_0.53_Ga_0.47_As structure.

In_0.53_Ga_0.47_As cell	Thickness (µm)		Doping concentration (cm^−3^)	
	Lower limit (*x*_*L*_)	Upper limit (*x*_*U*_)	Lower limit (*x*_*L*_)	Upper limit (*x*_*U*_)
Cap layer	0.02	0.14	8 × 10^17^	6 × 10^19^
FSF layer	0.02	0.14	8 × 10^17^	6 × 10^19^
Emitter layer	0.05	0.32	5 × 10^16^	7 × 10^18^
Base layer	2.00	20.0	5 × 10^16^	1 × 10^18^
BSF Layer	0.02	0.14	8 × 10^17^	6 × 10^19^
Buffer layer	0.02	0.14	8 × 10^17^	6 × 10^19^

*Q*_*emit*_ is the thermal emission of the blackbody at a fixed temperature (*T*_*BB*_), and the *Q*_*filter*_ is the thermal reflected emission of the low pass optical filter. The photon flux of an emitting blackbody, *Φ*, as a function of the emitted *λ* and *T*_*BB*_, is calculated via Planck’s Law in Eq. (4).

4 $$\Phi (\lambda ,T_{BB} ) = \frac{{2\pi hc_{o}^{2} }}{{\lambda^{2} (\exp (\frac{{hc_{o} }}{{k_{B} \lambda T_{BB} }}) - 1)}}$$

where *h* is the Planck’s constant, *k*_*B*_ is the Boltzmann’s constant and *c*_*o*_ is the speed of light.

The *P*_*out*_ of the cell is defined as:

5 $$P_{out} = V_{m} J_{m}$$

where the photocurrent, *J*_*m*_, as a function of voltage across the cell, *V*_*m*_, is the difference between the short circuit current and recombination loss, given by:

6 $$J_{m} = J_{SC} - (R_{rad} + R_{SRH} + R_{Aug} )$$

where *R*_*rad*_, *R*_*SRH*_ and *R*_*Aug*_, are the radiative, SRH and Auger recombination rates, respectively.

Based on the principle of detailed balance, the *J*_*sc*_ is calculated from the external quantum efficiency *EQE*(*λ*) and the *Φ*(*λ*):

7 $$J_{SC} = e\int\limits_{0}^{{\lambda_{{E_{g} }} }} {\Phi (\lambda )} EQE(\lambda )d$$

The fitness ratio is defined as the efficiency ratio (*η*_*r*_*(X)*), and it is shown in Eq. (8). The chromosomes are arranged based on their fitness from higher to lower (*i*^*`*^ = 1–50), and then some evolution mechanisms like survivor selection, crossover and mutation were used to build the next generation (Gen) using Excel and Visual basic. A 20% (10-best fitted chromosomes: *X*_*i*_ = *X*_*1*_ to *X*_*10*_) of the best chromosomes survived and directly passed to the next Gen as ${X}_{{i}^{^{\prime}}}$ while 80% (40-best fitted chromosomes) of the best-selected chromosomes go to the next step of crossover and mutation producing a new set of population^68^. This process is presented in Eq. (9).

8 $$\eta_{r} (X) = \left| {\frac{{\eta_{r} (X_{i} )}}{{\Sigma_{i = 1}^{50} \eta (X_{i} )}}} \right|$$

9 $$\left\{ {\begin{array}{*{20}c} {} \\ {} \\ {} \\ \end{array} } \right.\begin{array}{*{20}c} {X_{i} = X_{{i^{\prime}}} } \\ {p_{c} = 0.8,p_{m} = 0.01} \\ {X_{i} = 0} \\ \end{array} \begin{array}{*{20}c} {} & {} \\ \end{array} \begin{array}{*{20}c} {1 \le i^{\prime} \le 10} \\ {1 \le i^{\prime} \le 40} \\ {i^{\prime} > 40} \\ \end{array}$$

After some Gen’s simulations, child chromosomes were created from the best performing parent chromosomes producing the optimum In_0.53_Ga_0.47_As configuration. A stopping criterion was decided after observing no significant change in the efficiency and efficiency ratio within the Gen (*η*_r_(*X*) = 2 ± 0.3). In this way, the optimization process was repeated for illumination source temperatures from 800 to 2000 K, with an interval of 200 K.

## Results and discussion

### Single layer/variable optimization

The effect of varying the thickness and doping concentration of cap, FSF, emitter, base, BSF and buffer layers on the performance parameters (*J*_*sc*_, *V*_*oc*_, *FF*, *η*) are tabulated in Table 5. The variables are classified into three categories: insignificant where the variation in *η* is ≤ 0.4%, significant where the change in *η* is between 0.4 and 3% and highly significant where the change in *η* is ≥ 3%.

**Table 5:** The summary of the result for single variable optimization for blackbody temperature from 800 to 2000 K.

Designing variables	Optimum range	Output parameters				Designing variables
		*η*	*FF*	*V*_*oc*_	*J*_*sc*_	
Cap thickness	~ 0.02	**√**	**√**	**√**	** × **	Significant for all *T*_*BB*_
Cap doping	> 1 × 10^19^	**√**	**√**	** × **	** × **	Significant for *T*_*BB*_ > 1000 K
FSF thickness	< 0.09	**√**	**√**	**√**	** × **	Significant for *T*_*BB*_ < 1400 K
FSF doping	> 5 × 10^18^	** × **	** × **	** × **	** × **	Insignificant for all *T*_*BB*_
Emitter thickness	0.1–0.16	**√**	**√**	**√**	**√**	Significant for all *T*_*BB*_
Emitter doping	2–3 × 10^17^	**√**	**√**	**√**	** × **	Significant for *T*_*BB*_ < 1400 K
Base thickness	5–16	**√**	**√**	**√**	**√**	Highly significant for all *T*_*BB*_
Base doping	3–6 × 10^17^	**√**	**√**	**√**	**√**	Significant for all *T*_*BB*_
BSF thickness	~ 0.025	**√**	**√**	** × **	**√**	Highly significant at 2000 K and significant for 1600 K ≤ *T*_*BB*_ < 1800 K
BSF doping	~ 1 × 10^18^	** × **	** × **	** × **	** × **	Insignificant for all *T*_*BB*_
Buffer thickness	< 0.4	**√**	**√**	**√**	** × **	Significant for all *T*_*BB*_
Buffer doping	** > **1 × 10^19^	**√**	**√**	** × **	** × **	Significant and more significant for *T*_*BB*_ > 800 K

*√ Changing the variable affects the output parameter (percentage improvement by ±  ≥ 1%) and * × Changing the variable does not affect the output parameter (percentage improvement by ±  < 1%).

As can be seen from Table 5, the *V*_*oc*_ is not significantly affected by the doping concentration of the cap, FSF, BSF and buffer layers. Theoretically, *V*_*oc*_ is influenced by dark current densities (*J*_*01*_ and *J*_*02*_), where *J*_*01*_ is contributed to the dark current due to surface and bulk recombination losses, and *J*_*02*_ is related to recombination due to traps in the space charge region (SCR)^69^. However, the implementation of InP in the front and rear side of the junction reduces the front surface recombination and back surface recombination, eliminating the dark current across the surface. The variation of cap, FSF, BSF and buffer doping concentration produces no effect on the *V*_*oc*_ of the cell since no absorption and recombination have occurred. This is because the Type-II band alignment (staggered band) between InP/In_0.53_Ga_0.47_As and In_0.53_Ga_0.47_As /InP led to spatial separation of electrons and holes and passivated the surfaces^70^. However, the thickness increment of those layers will affect the *V*_*oc*_ because of the losses due to the light aborption^71^.

The *J*_*sc*_ is mainly related to the absorption and diffusion length of the photo-generated carriers, as shown in Eq. (10).^72^.

10 $$I_{sc} = qG(L_{n} + L_{p} )$$

where *G* is the generation rate, *q* is the charge, and *L*_*n*_ and *L*_*p*_ are the electron and hole diffusion length, respectively.

Since In_0.53_Ga_0.47_As material has a long electron and hole diffusion lengths, the increment of emitter, base and BSF thicknesses will significantly improve the absorption, carrier’s generation and *J*_*sc*_. For instance, the generated electrons (holes) at absorber layers has a diffusion length of 18.89 (5.88) µm at 1 × 10^17^ cm^−3^ doping concentration. The increment of base layer thickness from 1 to 18.89 µm will increase the *J*_*sc*_ since majority of the generated minority carriers (electrons) are able to reach the SCR before they are recombined. The doping concentrations of the absorber (emitter, base and BSF layers) affect the probability of carrier recombination as the mobility, lifetime and diffusion length of carriers decrease with higher doping concentration. However, since the emitter layer and BSF layer were remained constant at 0.05 µm and 0.025 µm, the variation of doping concentration gives a minor effect on the cell *J*_*sc*_.

The *FF* is defined as the ratio between the *V*_*oc*_*I*_*sc*_ to the actual operating condition of the cell *P*_*mp*_ = *I*_*mp*_*V*_*mp*_ after considering the series resistance and shunt resistance in the structure^73^. The *P*_*mp*_, *I*_*mp*_ and *V*_*mp*_ are denoted as the cell maximum power, maximum current and maximum voltage, respectively. It can be seen that the *FF* and *η* performance parameters were affected by almost all of the design variables. This is due to the domination of resistance losses when manipulating the variable individually. Nevertheless, the manipulation of FSF and BSF doping concentration had no significant impact on cell performance since the thickness of those layers are kept at very thin (~ 0.02 µm), resulting in lower resistance losses and minor absorption.

**Figure 3:**
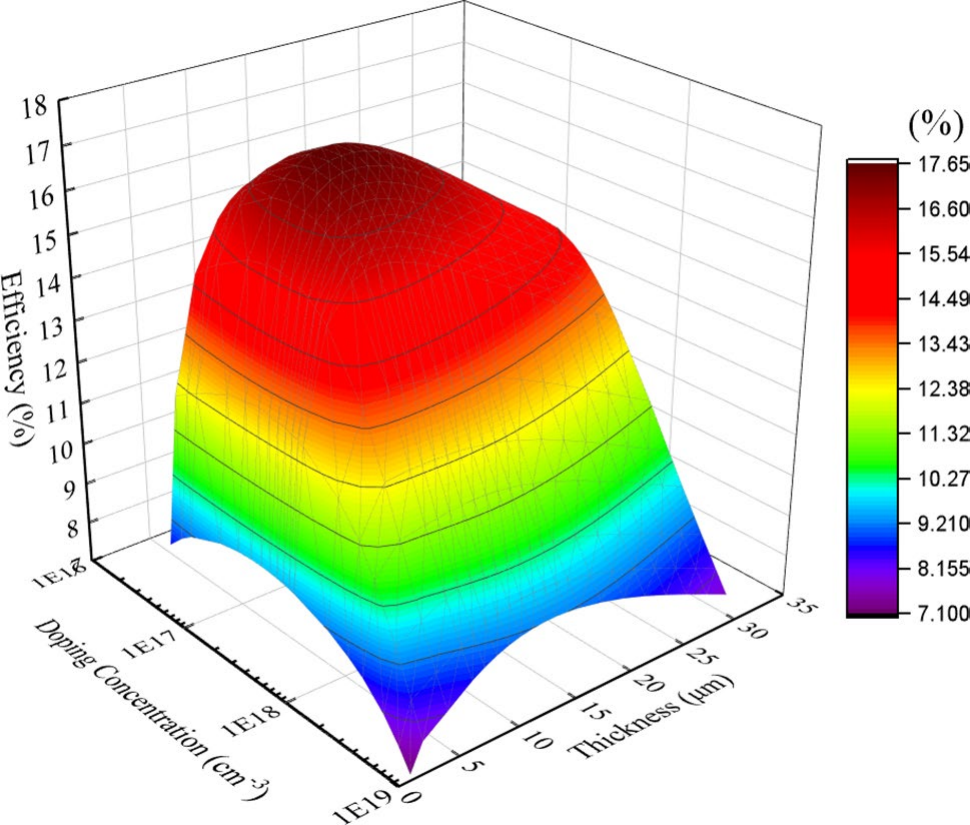
The efficiency of In_0.53_Ga_0.47_As TPV cell vs thickness and doping concentration of the base layer under 1400 K blackbody temperature.

Based on the single variable optimization, it was found that the manipulation of the thickness and doping concentration variables for the base layer significantly affects all performance parameters. In particular, the highest simulated *η* is achieved at an optimum base layer thicknesses of 5 and 16 µm with a blackbody temperature of 2000 K and 800 K, respectively. The variation of the optimal base thicknesses is related to the radiation spectrum and its peak wavelength (*λ*_*p*_). Based on Wien’s displacement law, *λ*_*p*_ of the spectrum shifts toward shorter IRs for higher blackbody temperature hence thinner base layer is required. Low doping in the base layer led to a high minority carrier lifetime and long diffusion length, which could increase the probability of carriers reaching the contact before recombining. However, it could also reduce the conductivity of the layer, electric field, and the built-in potential at the junction that decreases the *V*_*oc*_ performance, offsetting the improvement in *J*_*sc*_. On the other hand, a thicker base layer will increase the absorption of IRs, which results in higher *J*_*sc*_. However, it could also increase the recombination and shunt resistance^74,75^. Increasing the thickness is detrimental to cell performance since it causes higher recombination, especially if the structure has high SRH rate^76^. Moreover, the minority carriers generated in the lower region of the cell with diffusion length shorter than the thickness has a higher probability of recombining before reaching the SCR^74^. Therefore, it is worth exploring the tradeoff relationship of the base layer thicknesses and doping concentrations to acquire the optimum configuration of the base layer that produces the highest cell efficiency. The correlation of cell efficiency with the base layer thickness and base doping concentration is depicted in Fig. 3. The base thickness (doping concentration) was varied between 1 (6 × 10^16^) and 28 µm (1 × 10^19^ cm^−3^), while the rest of structure design variables were kept at their baseline values.

Based on Fig. 3, it was found that an optimum *η* of 17.61% is obtained at an optimum base thickness (doping concentration) of 11 µm (1 × 10^17^ cm^−3^). The increment in base thickness improves the absorption of IR, especially the band-edge photons^76^. Based on the Einstein relationship, Caughey-Thomas model and SRH model, the diffusion length of electron and hole are calculated as a function of doping concentration. At base doping of 1 × 10^17^ cm^−3^, the diffusion length of electron (hole) is equal to 18.89 (5.88) µm. The electron diffusion length of 18.89 µm is longer than the 11 µm base thickness. Since the diffusion length is longer than the base thickness, the probability that photo-generated carriers can reach the SCR is quite high^74^. Furthermore, the high diffusion coefficient of In_0.53_Ga_0.47_As carriers permits the formation of the active junction at low doping concentration. In_0.53_Ga_0.47_As cell ability to produce active junction at low doping concentration allows it to obtained high cell efficiency with minimum recombination rate.

### Multi-dimensional optimization using real coded genetic algorithm method

The results of multi-dimensional optimization on In_0.53_Ga_0.47_As TPV cell model under 800–2000 K blackbody temperatures are summarized in Table 6. It was found that the optimized cell presented in this work has thicker base layer as compared to those TPV cells reported in Table 1. At 1800 K blackbody temperature, the optimized cell has an efficiency of 20.48%, compared to 15% reported for TPV cell tested under similar testing conditions^40^. Compared to the reported In_0.53_Ga_0.47_As TPV cell efficiencies which can be as high as 30%^32,33,77^, the optimized structure in this paper does not consider photons recycling and ARC. Instead of utilizing photon recycling, this work focuses on the TPV cell design structure. A thicker base layer between 16 and 18 µm is preferred to enhance the current density as it increases the absorption of near band-edge photons and free-carrier absorption (FCA), which highly impact TPV cell efficiency^78,79^. At base layer thickness of 16–18 µm, the maximum cell efficiency of 23.18% was reported at 1400 K radiation temperature, while the minimum efficiency of 18.41% was achieved at 800 K. The electrical losses due to the high photogenerated carriers increased at radiation temperatures > 1400 K, which reduced the *η*. Although maximum efficiencies were achieved with base layer thickness of 16–18 µm, considering the technical difficulties and cost in growing high-quality In_0.53_Ga_0.47_As layer at > 10 µm, the base layer thickness was reduced to 8 µm. With this reduction in base layer thickness, the efficiency of the TPV cell is reduced only by an average of 1% (Maximum reduction of 1.9% at 1000 K and minimum reduction of 0.1% at 2000 K). RCGA_practical_ represents the RCGA results after taking into account the practicality in the growth of semiconductor wafer by reducing the base layer thickness to 8 µm. A thinner cap and buffer layers are designed with a higher doping concentration to form a better front and rear ohmic contacts, resulting in higher *FF* and lower series resistance. Besides, a thin FSF and BSF layers are designed with a higher doping concentration, resulting in a better front and rear junction passivation with minimum optical absorption.

**Table 6:** The optimum In_0.53_Ga_0.47_As TPV configuration from real coded genetic algorithm method (RCGA_practical_).

*T*_*BB*_ (K)	In_0.53_Ga_0.47_As cell												Performance			
	Cap layer		FSF layer		Emitter layer		Base layer		BSF layer		Buffer layer		*V*_*oc*_ (V)	*J*_*sc*_ (mA/cm^2^)	*FF* (%)	*η* (%)
	Thickness (µm)	Doping (cm^3^)	Thickness (µm)	Doping (cm^3^)	Thickness (µm)	Doping (cm^3^)	Thickness (µm)	Doping (cm^3^)	Thickness (µm)	Doping (cm^3^)	Thickness (µm)	Doping (cm^3^)				
800	0.05	1 × 10^19^	0.06	1 × 10^19^	0.28	2 × 10^17^	8	2.5 × 10^17^	0.05	1 × 10^19^	0.03	7 × 10^18^	0.34	13.01	73.85	16.73
1000	0.02	1 × 10^19^	0.04	6.5 × 10^18^	0.15	2 × 10^17^	8	2.5 × 10^17^	0.06	1 × 10^19^	0.02	9 × 10^18^	0.40	127.25	75.03	20.07
1200	0.02	1 × 10^19^	0.03	7.5 × 10^18^	0.15	2 × 10^17^	8	2.5 × 10^17^	0.03	1 × 10^19^	0.02	7 × 10^18^	0.44	564.74	72.29	21.63
1400	0.02	8.5 × 10^18^	0.04	9 × 10^18^	0.15	3 × 10^17^	8	2 × 10^17^	0.04	8.5 × 10^18^	0.02	9.5 × 10^18^	0.46	1719.35	68.63	22.06
1600	0.02	2 × 10^19^	0.02	5.5 × 10^18^	0.11	3 × 10^17^	8	2.5 × 10^17^	0.02	2 × 10^19^	0.02	9 × 10^18^	0.49	4064.67	64.73	21.74
1800	0.03	7 × 10^18^	0.02	9 × 10^18^	0.13	2 × 10^17^	8	2.5 × 10^17^	0.03	7 × 10^18^	0.02	9 × 10^18^	0.51	8201.45	59.22	20.48
2000	0.02	8.5 × 10^18^	0.03	1 × 10^19^	0.13	2 × 10^17^	8	4 × 10^17^	0.03	9.5 × 10^18^	0.02	8 × 10^18^	0.52	14,529.30	56.61	19.76

A proper cell design has to consider both optical and electronic losses. The optical losses were the dominant factor in determining the change in efficiencies of the non-optimized cell. Optimized cell efficiency, on the other hand, were dominated by the electrical losses, especially at high radiation temperature with high energy spectrum density. The In_0.53_Ga_0.47_As TPV cell has to be optically thick (i.e. to absorb all or most of the incident illumination) and electronically thin (i.e. to collect the photoexcited electron–hole pairs with little or no losses). These two requirements lead to an optimal configuration that maximizes efficiency. At 1400 K blackbody temperature, *J*_*sc*_ and *FF* are increased respectively from 755.01 to 1719.35 mA/cm^2^ and 62.65 to 68.63% after optimizing the entire cell configuration. Most of the optimization works focused on optimizing the electrical losses of cell to improve the *V*_*oc*_ performance by reducing the thickness of the absorber layer^27,80^. However, the absorption of near band-edge photons is neglected. Alharbi et al.^81^ highlighted that the leading cause of the reduced efficiency of a solar cell below the theoretical limit is the drop in the estimated *V*_*oc*_ while usually, the obtained *J*_*sc*_ is around the theoretically maximum values. It is important to mention that the solar spectrum is mainly concentrated around the visible region, and these photons do not require thicker base absorber. On the other hand, TPV illumination flux is usually concentrated at infrared wavelengths, and thicker absorber is needed to improve the absorption of IRs and significantly increase *J*_*sc*_. This statement was supported by the optimization study reported by Baudrit and Algora^46^, where increasing the absorber thickness of the bottom cell in GaInP/GaAs dual-junction increases the value of photocurrent density. Furthermore, the *J*_*sc*_ and *η* were increased respectively from 13.85 to 15.62 A/cm^2^ and 32.6 to 36.4% under 1000 suns concentration.

For the validation of optimization, the In_0.53_Ga_0.47_As TPV structure reported from the RCGA optimization method was validated by two validation methods (VM), which are single-variable method (VM 1) and iteration method (VM 2). VM 1 utilized the results from the single-variable optimization where the optimum values of the 12 design parameters obtained through single-variable optimization were used to determine the efficiency of the TPV cell. VM 2 utilized the iteration method to simulate all possibilities for the 12 design parameters. However, it was estimated that the total number of iterations are 12^10^ simulation runs. To reduce the computation time to a manageable level, we used a bigger step and only varied the important design variables while the insignificant variables were maintained at their baseline values. Due to these reasons, it is expected that the cell efficiencies obtained through VM 1 and VM 2 are slightly lower than those obtained through RCGA method.

The results obtained through RCGA, VM 1 and VM 2 are tabulated in Table 7. For different radiation temperatures, the average percentage differences between RCGA and VM 1, and between RCGA and VM 2, were 5.17% and 6.95%, respectively. On the other hand, RCGA method is able to converge to the optimum structure in a faster manner as compared to the long simulation time needed by the iteration method.

**Table 7:** The summary of the results obtained from RCGA, VM 1 and VM 2 with their percentage differences.

Radiation temperature	*η* using RCGA method (%)	Validation method 1		Validation method 2	
		*η* using single variable (%)	Percentage difference (%)	*η* using Iterations method (%)	Percentage difference (%)
800 K	18.4	17.44	5.22	17	7.61
1000 K	21.92	20.18	7.94	20.2	7.85
1200 K	23.1	22	4.76	21.5	6.93
1400 K	23.18	22	5.09	21.4	7.68
1600 K	22.46	21.35	4.94	20.7	7.84
1800 K	20.83	19.96	4.18	19.5	6.39
2000 K	19.76	18.96	4.05	18.9	4.35
Average	21.37	20.27	5.17	19.89	6.95

**Figure 4:**
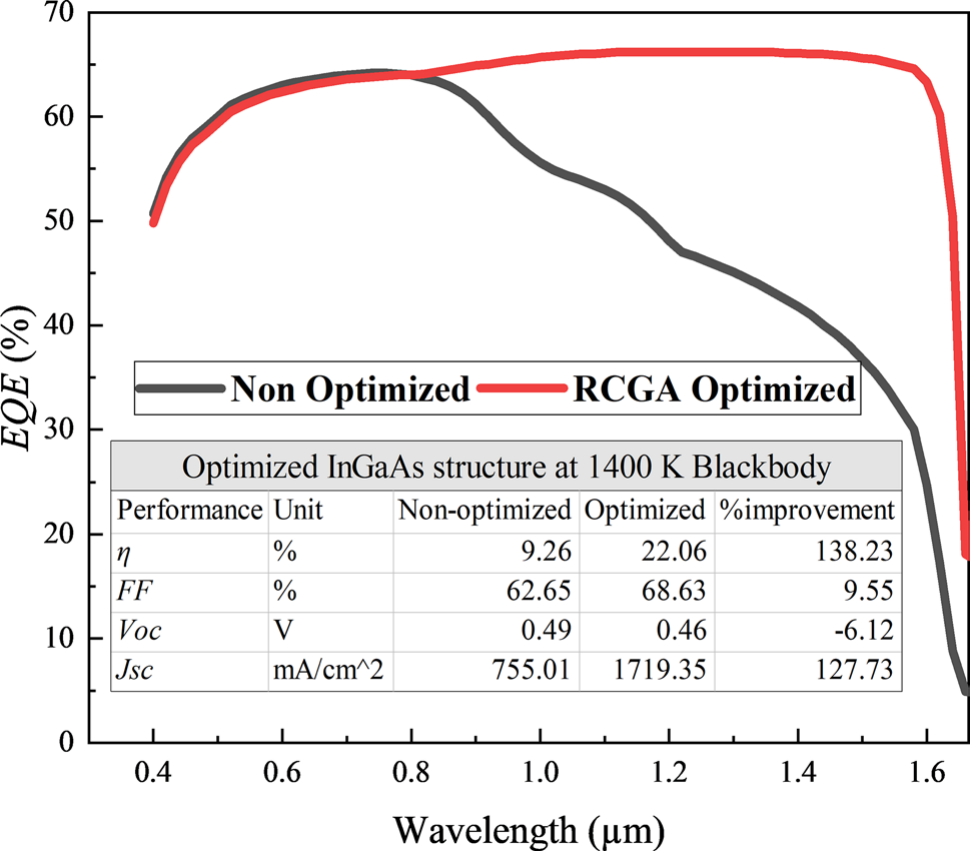
The external quantum efficiency vs wavelength for non-optimized and RCGA_practical_ optimized In_0.53_Ga_0.47_As TPV cell under 1400 K blackbody temperature.

Figure 4 illustrates the *EQE* of non-optimized and RCGA_practical_-optimized In_0.53_Ga_0.47_As TPV cells as a function photon λ. It can be seen from Fig. 4 that the *EQE(*λ) of the multi-variable optimized cell is higher than that of the non-optimized TPV cell. This is due to the enhancement of photocurrent generation at a wavelength higher than 0.85 µm. A possible explanation for this is that the optimized In_0.53_Ga_0.47_As TPV configuration tends to have higher absorption and collection of IRs photo-generated carriers. Despite the reduction in *V*_*oc*_ by 6.12% after the optimization process, *J*_*sc*_ is significantly increased by 127.73%, resulted in the improvement in the TPV cell efficiency by 138.23%.

Based on the optimized results, a significant increase in *η* is attained when the base layer increased from 1 to 8 µm. This finding can be supported by an analysis of the absorption coefficient and the absorption length of the In_0.53_Ga_0.47_As cell. The absorption coefficient describes the light penetration in a semiconductor before being absorbed and can be obtained using Kramers–Kronig Dispersion relation as follows:

11 $$\alpha (\lambda ) = \frac{4\pi k(\lambda )}{\lambda }(\mu {\text{m}}^{ - 1} )$$

where *k(λ)* is the extinction coefficient of In_0.53_Ga_0.47_As^82,83^. On the other hand, the absorption length (*α*^−*1*^) is given as the inverse of the *α* and describes the penetration length of majority photons in semiconductor before being absorbed. Due to absorption in the material, the illumination intensity weakened with increasing penetration length and can be described by means of a decaying exponential function, as shown in Eq. (12).^74^.

12 $$\alpha_{Abs} = (1 - R).(1 - e^{ - \alpha d} )$$

where *η*_*Abs*_ is absorption efficiency, the effective cell thickness (*d*) can be determined from Eq. (13) by maximizing the absorption in the cell *η*_*Abs*_ =  ~ 99% and *R* = 0^74^.

13 $$0.99 = (1 - e^{ - \alpha d} ) = 1 - 0.99 \to d = \frac{\ln (1 - 0.99)}{{ - \alpha }}\mu {\text{m}}$$

**Figure 5:**
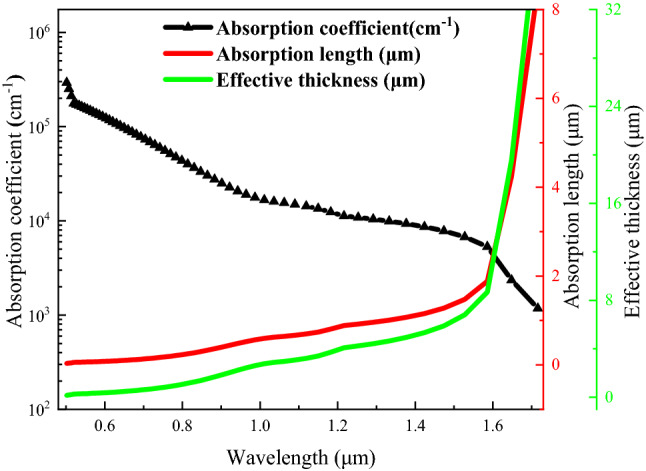
The absorption coefficient, absorption length and effective thickness at different wavelengths.

Figure 5 shows the result of the *α*, *α*^−1^and *d* of In_0.53_Ga_0.47_As as a function of λ. It can be seen that thicker absorber layer is needed to absorb IRs. For instance, photons with λ of 1.65 μm have an absorption length of 4.25 μm. To effectively absorb ~ 99% of the photons, the cell thickness will therefore be approximately 19.59 μm.

The In_0.53_Ga_0.47_As has a spectral response with wavelength at around 1.75 μm. It can be seen that infrared light requires a thick absorber, so that majority of the photons are absorbed. The illumination intensity of the blackbody is mainly concentrated at λ > 1.0 μm. For instance, 1400 K blackbody temperature has 69.25% of power density for wavelengths between 1 and 1.8 μm as compared to 3.43% of power density for wavelengths between 0.2 and 1 µm. An optical BSR is used to improve the light absorption, as the optical path distance can be doubled due to the back reflection^20^. Therefore, although the effective thickness to absorb photons up to 1.75 μm is approximately 32–36 μm (based on Fig. 5), the effective device thickness can be reduced to approximately 16–18 μm. However, based on additional simulation work upon completing the multi-dimensional optimization, an effective thickness higher than 8 μm (up to 18 μm) will only increase the efficiency by an average of about 1% for various blackbody temperatures due to the sharp decline of the absorption coefficient for λ ≥ 1.63 µm. Other than that, the light trapping method such as Lambertian rear reflector and textured surface can be used to improve the light absorption in the cell^74,84^.

A further explanation for the high *EQE* is due to the effective separation and collection of generated carriers. In_0.53_Ga_0.47_As structure is frequently constructed with FSF and BSF layers to reduce the surface recombination and enhance the *V*_*oc*_^41,85^. High to low doping concentration between the FSF or BSF and the active junction is vital to generate a SCR similar to the SCR between n-p junction. For instance, holes diffused out of this highly doped BSF layer into the lower-doped base layer, leaving site-fixed negatively charged acceptor atoms behind^74^. The generated electrical field that acts like an electric mirror that returns the electrons generated through absorption in the direction of the SCR^74^. The probability of undesired recombination at the rear of the cell is thus significantly reduced. Belghachi et al.^86^ described a mathematical model on the importance of high-low junction in the front and rear sides of GaAs cell, which play a crucial role in enhancing the light-generated free carriers’ collection. The thickness of FSF and BSF layers should be as thin as possible while the doping concentration should be as high as 1 × 10^19^ cm^−3^^86^. The band diagram is an alternative way to view the effect of FSF and BSF layers. The band diagram of the In_0.53_Ga_0.47_As/InP cell was extracted from TCAD model, with the FSF and BSF layers.

**Figure 6:**
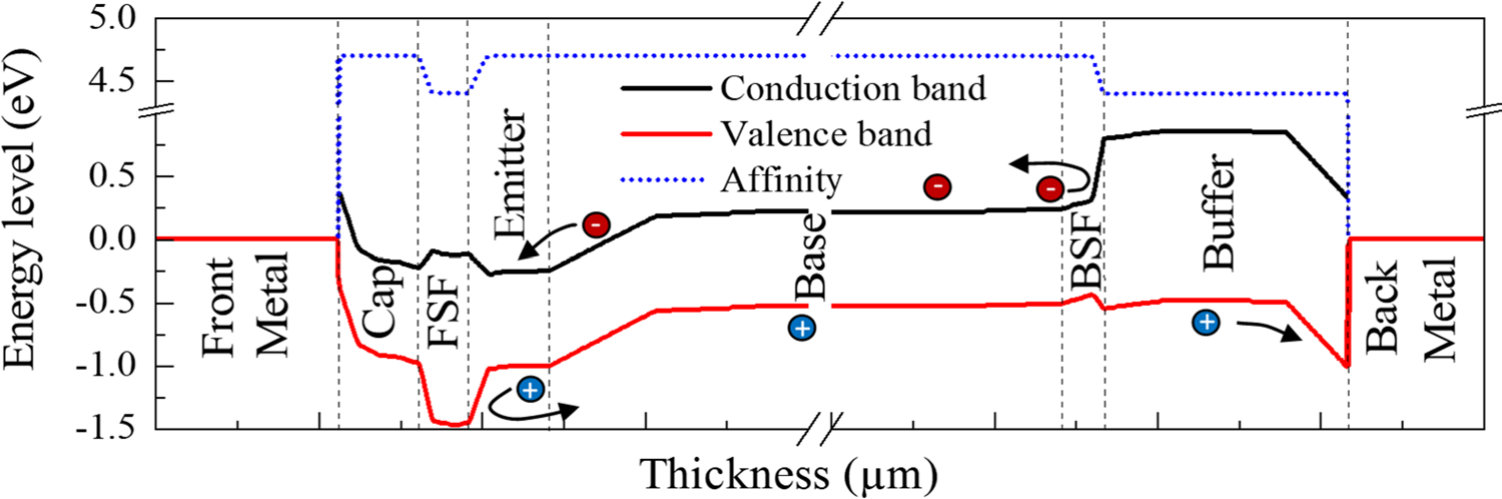
The band diagram of In_0.53_Ga_0.47_As cell heterojunction.

Figure 6 presents the band diagram of In_0.53_Ga_0.47_As heterojunction. The band offset between In_0.53_Ga_0.47_As and InP forms type–II band (staggered) leads to spatial separation of electrons and holes. A discontinuity in conduction for base/(BSF/buffer) interface prevents the electrons from moving further up to the back contact while allowing the flow of holes. The discontinuity in valance for the FSF/emitter interface prevents the holes from moving further up to the front contact while allowing the flow of electrons^74^. The band alignment of In_0.53_Ga_0.47_As structure was investigated in several studies^87,88^. The n-InP will form a barrier to holes, but a sink for electrons at the FSF and visa verse at the BSF/buffer. Besides that, cell total voltage is now divided into the potential level at n-p junction and additional level at n^+^n and pp^+^ interfaces^74^. Hence, FSF layer improves the *EQE* of shorter wavelengths photo-generated carriers, and the BSF/buffer layer increases the *EQE* of longer wavelengths photo-generated carriers^86^. It should be stressed that BSF/buffer layer is very significant to enhance the *J*_*sc*_ and *V*_*oc*_ of the TPV cell since the long-wavelength photons tend to absorb at the deeper region of the structure. The combination of both thicker absorber layer and BSF/buffer significantly improves the collection of photocurrent collection of *λ* near to the band-edge of In_0.53_Ga_0.47_As TPV cell.

## Conclusion

In summary, the simulation model is validated with reported experimental data, generating a low *η* percentage error of 0.61% between experimental and simulation. Research gap was identified based on comprehensive comparison of previous structure designs and performance of In_0.53_Ga_0.47_As cell. Single variable and multi-dimensional optimization (RCGA) of heterojunction In_0.53_Ga_0.47_As cell are developed and applied at 800–2000 K TPV radiation temperatures. The single variable optimization is used to investigate the effect of thickness and doping concentration of layers, which demonstrated the significant impact of the base layer to achieve high performance In_0.53_Ga_0.47_As TPV cell. Meanwhile, under radiation temperatures ranging from 800 to 2000 K, optimized In_0.53_Ga_0.47_As heterojunction TPV cell using RCGA increases the *η* by an average of 11.86% as compared to the reference structure. It was found that the incorporation of a thicker absorber with effective barrier layer BSF/Buffer layer improves the absorption and collection of photo-generated carriers near the band-edge, which produced higher output performance. The increment of the In_0.53_Ga_0.47_As cell efficiency leads to a significant increase in the generated output power, demonstrating great potential of TPV for industrial waste heat harvesting. Finally, the method of hybridizing the Silvaco TCAD software with RCGA for multi-dimensional optimization can be readily adopted in optimization work for semiconductor devices, such as solar cell, TPV cell and photodetectors.

## References

[1] Wang X, Liang R, Fisher P, Chan W, Xu J (2020). Radioisotope thermophotovoltaic generator design methods and performance estimates for space missions. J. Propuls. Power.

[2] Fraas, L. M. *Thermophotovoltaics Using Infrared Sensitive Cells in Low-Cost Solar Electric Power*. *Springer, Cham* (Springer International Publishing, 2014). 10.1007/978-3-319-07530-3.

[3] Fraas L. M., Avery, J. E., Huang, H. X., & Martinelli R. U. Thermophotovoltaic system configurations and spectral control. *Semicond. Sci. Technol.* **18**, S165–S173 (2003).

[4] Schock A, Or C, Kumar V (1997). Design and integration of small RTPV generators with new millennium spacecraft for outer solar system. Acta Astronaut..

[5] Krier A, Yin M, Marshall ARJ, Krier SE (2016). Low bandgap InAs-based thermophotovoltaic cells for heat-electricity conversion. J. Electron. Mater..

[6] Seal M, Christ S, Campbell G, West E, Fraas L (1997). Thermophotovoltaic generation of power for use in a series hybrid vehicle. SAE Technical Papers.

[7] Lenert A (2014). A nanophotonic solar thermophotovoltaic device. Nat. Nanotechnol..

[8] Elzouka M, Ndao S (2017). Towards a near-field concentrated solar thermophotovoltaic microsystem: Part I Modeling. Sol. Energy.

[9] A. Datas, C. A. Development and experimental evaluation of a complete solar thermophotovoltaic system. *Prog. PHOTOVOLTAICS Res. Appl.* **15**, 326–334 (2012).

[10] Amy C, Seyf HR, Steiner MA, Friedman DJ, Henry A (2019). Thermal energy grid storage using multi-junction photovoltaics. Energy Environ. Sci..

[11] Seyf HR, Henry A (2016). Thermophotovoltaics: A potential pathway to high efficiency concentrated solar power. Energy Environ. Sci..

[12] Utlu Z, Önal BS (2018). Thermodynamic analysis of thermophotovoltaic systems used in waste heat recovery systems: An application. Int. J. Low-Carbon Technol..

[13] Fraas, L. M. Economic potential for thermophotovoltaic electric power generation in the steel industry. in *2014 IEEE 40th Photovoltaic Specialist Conference, PVSC 2014* 766–770 (2014). 10.1109/PVSC.2014.6925031.

[14] Shan S, Zhou Z, Cen K (2019). An innovative integrated system concept between oxy-fuel thermo-photovoltaic device and a Brayton-Rankine combined cycle and its preliminary thermodynamic analysis. Energy Convers. Manag..

[15] Rashid, W. E. S. W. A. *et al.* Recent development of thermophotovoltaic system for waste heat harvesting application and potential implementation in thermal power plant. *IEEE Access* **8**, 105156–105168 (2020).

[16] Yang Z (2017). An efficient method exploiting the waste heat from a direct carbon fuel cell by means of a thermophotovoltaic cell. Energy Convers. Manag..

[17] Van der Heide J, Posthuma NE, Flamand G, Geens W, Poortmans J (2009). Cost-efficient thermophotovoltaic cells based on germanium substrates. Sol. Energy Mater. Sol. Cells.

[18] Lotfi H (2017). Narrow-bandgap interband cascade thermophotovoltaic cells. IEEE J. Photovoltaics.

[19] Bett AW, Sulima OV (2003). GaSb photovoltaic cells for applications in TPV generators. Semicond. Sci. Technol..

[20] Burger T, Fan D, Lee K, Forrest SR, Lenert A (2018). Thin-film architectures with high spectral selectivity for thermophotovoltaic cells. ACS Photonics.

[21] Hitchcock CW, Gutmann RJ, Borrego JM (1999). Antimonide-based devices for thermophotovoltaic applications. IEEE Trans. Electron Devices.

[22] Choi HK (1997). High-performance GaInAsSb thermophotovoltaic devices with an AlGaAsSb window. Appl. Phys. Lett..

[23] Mauk MG, Tata AN, Cox JA (2001). Solution growth of thick III-V antimonide alloy epilayers (InAsSb, InGaSb, InGaAsSb, AlGaAsSb, and InAsSbP) for ‘virtual substrates’. J. Cryst. Growth.

[24] Bitnar B, Durisch W, Holzner R (2013). Thermophotovoltaics on the move to applications. Appl. Energy.

[25] Bauer, T. *Thermophotovoltaics*. *Green Energy and Technology* vol. 7 (Springer Berlin Heidelberg, 2011).

[26] Kao Y-C (2019). Performance comparison of III–V//Si and III–V//InGaAs multi-junction solar cells fabricated by the combination of mechanical stacking and wire bonding. Sci. Rep..

[27] Sodabanlu, H., Watanabe, K., Sugiyama, M. & Nakano, Y. Growth of InGaAs(P) in planetary metalorganic vapor phase epitaxy reactor using tertiarybutylarsine and tertiarybutylphosphine for photovoltaic applications. *Jpn. J. Appl. Phys.* **57**, 08RD09 (2018).

[28] Yamada, T. *et al.* 5 × 5 cm 2 GaAs and GaInAs solar cells with high conversion efficiency. *Japanese J. Appl. Physics, Part 2 Lett.* **44**, 7–10 (2005).

[29] Wilt DM (1994). High efficiency indium gallium arsenide photovoltaic devices for thermophotovoltaic power systems. Appl. Phys. Lett..

[30] Zahler JM (2007). High efficiency InGaAs solar cells on Si by InP layer transfer. Appl. Phys. Lett..

[31] Dharmarasu N (2001). High-radiation-resistant InGaP, InGaAsP, and InGaAs solar cells for multijuction solar cells. Appl. Phys. Lett..

[32] Omair Z (2019). Ultraefficient thermophotovoltaic power conversion by band-edge spectral filtering. Proc. Natl. Acad. Sci..

[33] Fan D (2020). Near-perfect photon utilization in an air-bridge thermophotovoltaic cell. Nature.

[34] Crystals, J. http://jxcrystals.com/GaSb/4sale5.pdf. 1–2.

[35] Bauhuis, G. J., Mulder, P., Haverkamp, E. J., Huijben, J. C. C. M. & Schermer, J. J. 26.1% thin-film GaAs solar cell using epitaxial lift-off. *Sol. Energy Mater. Sol. Cells* **93**, 1488–1491 (2009).

[36] Emziane M, Nicholas RJ (2007). Optimization of InGaAs(P) photovoltaic cells lattice matched to InP. J. Appl. Phys..

[37] Tuley RS, Nicholas RJ (2010). Band gap dependent thermophotovoltaic device performance using the InGaAs and InGaAsP material system. J. Appl. Phys..

[38] Tan M (2014). Investigation of InGaAs thermophotovoltaic cells under blackbody radiation. Appl. Phys. Express.

[39] Tuley RS (2013). Lattice-matched InGaAs on InP thermophovoltaic cells. Semicond. Sci. Technol..

[40] Karlina, L. B., Vlasov, A. S., Kulagina, M. M. & Timoshina, N. K. Thermophotovoltaic cells based on In0.53Ga0.47As/InP heterostructures. *Semiconductors* **40**, 346–350 (2006).

[41] Kim C-Y, Cha J-H, Kim J, Kwon Y-S (2005). Open-circuit voltage improvement in InGaAs/InP heterojunction solar cells. Jpn. J. Appl. Phys..

[42] Matsubara H, Tanabe T, Moto A, Mine Y, Takagishi S (1998). Over 27% efficiency GaAs/InGaAs mechanically stacked solar cell. Sol. Energy Mater. Sol. Cells.

[43] Wilt, D. M. *et al.* Electrical and optical performance characteristics of p/n InGaAs monolithic interconnected modules. *NASA Lewis Res. Cent.* 1119–1124 (1997).

[44] Wojtczuk S, Gagnon E, Geoffroy L, Parodos T (1995). InxGa1−xAs thermophotovoltaic cell performance vs bandgap. AIP Conf. Proc..

[45] Baloch AAB (2017). Full space device optimization for solar cells. Sci. Rep..

[46] Baudrit M, Algora C (2010). Theoretical optimization of GaInP/GaAs dual-junction solar cell: Toward a 36% efficiency at 1000 suns. Phys. Status Solidi Appl. Mater. Sci..

[47] Jurczak P, Onno A, Sablon K, Liu H (2015). Efficiency of GaInAs thermophotovoltaic cells: The effects of incident radiation, light trapping and recombinations. Opt. Express.

[48] Salem AF, Brennan KF (1995). Theoretical study of the response of InGaAs metal-semiconductor-metal photodetectors. IEEE J. Quantum Electron..

[49] Levinshtein, M., Rumyantsev, S. & Shur, M. *Handbook Series on Semiconductor Parameters: Ternary And Quaternary III-V Compounds*. vol. 2 (World Scientific Publishing Co. Pte. Ltd., 1996).

[50] Datta S, Roenker KP, Cahay MM, Stanchina WE (1999). Implications of hole vs electron transport properties for high speed Pnp heterojunction bipolar transistors. Solid. State. Electron..

[51] Sotoodeh M, Khalid AH, Rezazadeh AA (2000). Empirical low-field mobility model for III-V compounds applicable in device simulation codes. J. Appl. Phys..

[52] Menon, P. S., Kandiah, K. & Shaari, S. Concentration and temperature-dependent low-field mobility model for in0.53Ga0.47As interdigitated lateral pin PD. *IEICE Electron. Express* **5**, 303–309 (2008).

[53] Ahrenkiel, R. K., Ellingson, R., Johnston, S. & Wanlass, M. Recombination lifetime of In0.53Ga0.47As as a function of doping density. *Appl. Phys. Lett.* **72**, 3470–3472 (1998).

[54] Zemel A, Gallant M (1995). Carrier lifetime in InP/InGaAs/InP by open-circuit voltage and photoluminescence decay. J. Appl. Phys..

[55] Chiang H, Rode JC, Choudhary P, Rodwell MJW (2014). Lateral carrier diffusion and current gain in terahertz InGaAs/InP double-heterojunction bipolar transistors. J. Appl. Phys..

[56] Cui D, Hubbard SM, Pavlidis D, Eisenbach A, Chelli C (2002). Impact of doping and MOCVD conditions on minority carrier lifetime of zinc- and carbon-doped InGaAs and its applications to zinc- and carbon-doped InP/InGaAs heterostructure bipolar transistors. Semicond. Sci. Technol..

[57] Zeng QY (2015). Dependence of dark current on carrier lifetime for InGaAs/InP avalanche photodiodes. Opt. Quantum Electron..

[58] SketchUp Free. *3D modeling computer program: Release 2020.0.* (Trimble Inc, Boulder, CO, 2017*).*

[59] Woolf DN (2018). High-efficiency thermophotovoltaic energy conversion enabled by a metamaterial selective emitter. Optica.

[60] Liu X (2011). Taming the blackbody with infrared metamaterials as selective thermal emitters. Phys. Rev. Lett..

[61] Tong JK, Hsu WC, Huang Y, Boriskina SV, Chen G (2015). Thin-film ‘thermal well’ emitters and absorbers for high-efficiency thermophotovoltaics. Sci. Rep..

[62] Dawoud B, Amer E, Gross D (2007). Experimental investigation of an adsorptive thermal energy storage. Int. J. energy Res..

[63] Fourspring PM, DePoy DM, Rahmlow TD, Lazo-Wasem JE, Gratrix EJ (2006). Optical coatings for thermophotovoltaic spectral control. Appl. Opt..

[64] Inoue T, Watanabe K, Asano T, Noda S (2018). Near-field thermophotovoltaic energy conversion using an intermediate transparent substrate. Opt. Express.

[65] Bernardi MP (2015). Impacts of propagating, frustrated and surface modes on radiative, electrical and thermal losses in nanoscale-gap thermophotovoltaic power generators. Sci. Rep..

[66] Chuang Y-C, Chen C-T, Hwang C (2016). A simple and efficient real-coded genetic algorithm for constrained optimization. Appl. Soft Comput..

[67] Liao T, Zhang H, Wang ZY (2020). Improved design of a thermophotovoltaic device. IEEE Trans. Electron Devices.

[68] Hassanat A (2019). Choosing mutation and crossover ratios for genetic algorithms—a review with a new dynamic approach. Information.

[69] Kayes, B. M. *et al.* 27.6% Conversion efficiency, a new record for single-junction solar cells under 1 sun illumination. in *2011 37th IEEE Photovoltaic Specialists Conference* vol. 28 000004–000008 (IEEE, 2011).

[70] Zhou H, Qu Y, Zeid T, Duan X (2012). Towards highly efficient photocatalysts using semiconductor nanoarchitectures. Energy Environ. Sci..

[71] Olmsted, N. & Record, C. InP solar cell with window layer. *United States Patent 19* (1994).

[72] Green, M. A. *Solar cells: operating principles, technology and system applications*. (University of New South Wales, 1986).

[73] Wu FL, Ou SL, Horng RH, Kao YC (2014). Improvement in separation rate of epitaxial lift-off by hydrophilic solvent for GaAs solar cell applications. Sol. Energy Mater. Sol. Cells.

[74] Mertens, K. Structure and method of operation of solar cells. in *Photovoltaics : Fundamentals, Technology and Practice* (2014). 10.1007/978-3-319-29650-0_7.

[75] Sun, Y., *et al.* Modeling wide bandgap GaInP photovoltaic cells for conversion efficiencies up to 16.5%. in *2015 IEEE 42nd Photovoltaic Specialist Conference (PVSC)* 1–6 (IEEE, 2015). 10.1109/PVSC.2015.7356074.

[76] Andreani, L. C., Bozzola, A., Kowalczewski, P., Liscidini, M. & Redorici, L. Silicon solar cells: Toward the efficiency limits. *Adv. Phys. X* **4**, (2019).

[77] Burger T, Sempere C, Roy-Layinde B, Lenert A (2020). Present efficiencies and future opportunities in thermophotovoltaics. Joule.

[78] Wilt D, Wehrer R, Palmisiano M, Wanlass M, Murray C (2003). Monolithic interconnected modules (MIMs) for thermophotovoltaic energy conversion. Semicond. Sci. Technol..

[79] Karalis A, Joannopoulos JD (2016). Squeezing’ near-field thermal emission for ultra-efficient high-power thermophotovoltaic conversion. Sci. Rep..

[80] Mathews, I. *et al.* InAlAs and InGaAs solar cell development for use in monolithic triple-junction solar cells with improved sprectrum splitting. in *28th European Photovoltaic Solar Energy Conference and Exhibition* (2013). doi:110.4229/28thEUPVSEC2013-1AV.2.40.

[81] Alharbi, F. H. *et al.* An efficient descriptor model for designing materials for solar cells. *NPJ Comput. Mater.* **1**, 15003 (2015).

[82] Adachi S (1989). Optical dispersion relations for GaP, GaAs, GaSb, InP, InAs, InSb, Al xGa1-xAs, and In1-xGaxAs yP1-y. J. Appl. Phys..

[83] Nee TW, Green AK (1990). Optical properties of InGaAs lattice-matched to InP. J. Appl. Phys..

[84] Kim Y, Lam ND, Kim K, Park W-K, Lee J (2017). Ge nanopillar solar cells epitaxially grown by metalorganic chemical vapor deposition. Sci. Rep..

[85] Gamel, M. *et al.* Effect of front-surface-field and back-surface-field on the performance of GaAs based-photovoltaic cell. in *2019 IEEE International Conference on Sensors and Nanotechnology* 1–4 (IEEE, 2019). 10.1109/SENSORSNANO44414.2019.8940098.

[86] Belghachi A, Helmaoui A (2008). Effect of the front surface field on GaAs solar cell photocurrent. Sol. Energy Mater. Sol. Cells.

[87] Loga R, Loga R, Vilches A (2004). Fabrication and characterization of circular geometry InP/InGaAs double heterojunction bipolar transistors. Semicond. Sci. Technol..

[88] Ginige, R., Cherkaoui, K., Wong Kwan, V., Kelleher, C. & Corbett, B. High injection and carrier pile-up in lattice matched InGaAs/InP PN diodes for thermophotovoltaic applications. *J. Appl. Phys.* **95**, 2809–2815 (2004).

